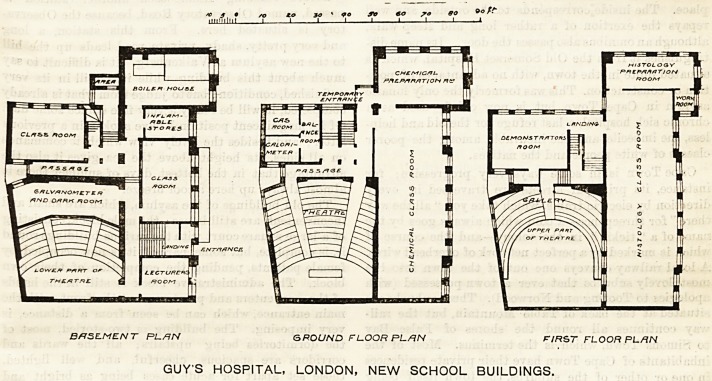# Hospital Construction

**Published:** 1897-07-10

**Authors:** 


### THE INSTITUTIONAL WORKSHOP.

### HOSPITAL CONSTRUCTION.

### NEW MEDICAL SCHOOL BUILDINGS, GUY'S HOSPITAL.

The new buildings which have recently been erected for the purposes of the Guy's Hospital Medical School form part of a scheme for providing under one roof all the accommodation required by the medical school, with the exception of the chemical, physical, public health, and bacteriological, recently erected in association with the dental school buildings. The part of the building which we now describe forms about one-third of the whole structure, and consists of the three lower floors of the south wing.

On the lower or half-basement floor are the lower part of the theatre, and in the part below the upper tiers of seats three dark rooms for the use of those engaged in chemical work. There are also certain class-rooms, a lecturer's room, store-rooms, and a boiler from which steam is supplied for heating the building. On the next floor there is an admirably-arranged chemical class-room, providing accommodation for 84 students, with a preparation room, which has been so fitted as to give ample space for six men carrying out original investigations. Attached to these are various subsidiary rooms, the whole being intended for the use of those studying physiological and pathological chemistry.

On the next floor the whole of the front is devoted to the laboratory and preparation room for normal and morbid histology. The laboratory is 80 ft. in length, and is fitted with four rows of benches running longitudinally, so arranged that all the students will face the light. These benches are provided with shafting for driving drums and other physiological apparatus. On the same floor is also a demonstrator's room specially adapted for experimental work, being provided with shafting and pulleys, worked by electro-motors, to drive the various kinds of recording apparatus employed. There is also a small workshop, fitted with lathe and carpenter's bench.

The theatre, which extends through all three floors, is extremely well arranged, in that every person can see the table. The gallery is constructed on the cantilever principle, so that there are no supporting columns to interfere with the view of those below. The lecturer's table is provided with water motor and shafting, and there is an arc light for lantern illustration. Altogether the theatre will hold from 400 to 500 men. The whole building is fireproof.

If we were critically inclined, we should draw attention to the meanness of the temporary entrances, and the narrowness of the staircases. It also seems an inconvenient arrangement that to reach the galvanometer and dark rooms, the balance, and the calorimeter rooms the students from the laboratories should have to cross the passages thronged by those coming from the theatre. We quite enter into the difficulty, however, of erecting a building piecemeal, and doubtless in the completed scheme the various parts will bear a proper relation to one another.

The architect is Mr. J. H. T. Woodd. The details of the construction of the gallery have been entrusted to Mr. Thomas Kirkland, the consulting engineer to the hospital, who has also supervised the heating and electric lighting of the building. The contract for the building was obtained by Messrs. Holloway, that for electric lighting by Messrs. Donnison and Co. The internal fittings have been constructed partly by Messrs. Hammer, and partly by Messrs. Hayward, of Manchester. The ironwork of the gallery was carried out by Messrs. Handyside, and the heating arrangements by Messrs. Jones and Attwood.

GUY'S HOSPITAL, LONDON, NEW SCHOOL BUILDINGS.

## Figures and Tables

**Figure f1:**